# Associations of cognitive activity and access to resources with cognitive decline in a broad representation of older adults

**DOI:** 10.1002/alz.71487

**Published:** 2026-05-21

**Authors:** Mayra L. Estrella, Maude Wagner, Robert S. Wilson, Lisa L. Barnes, David A. Bennett, David X. Marquez, Melissa Lamar

**Affiliations:** ^1^ Rush Alzheimer's Disease Center Rush University Medical Center Chicago Illinois USA; ^2^ Department of Internal Medicine Rush University Medical Center Chicago Illinois USA; ^3^ Department of Neurological Sciences Rush University Medical Center Chicago Illinois USA; ^4^ Department of Psychiatry and Behavioral Sciences Rush University Medical Center Chicago Illinois USA; ^5^ Department of Kinesiology and Nutrition University of Illinois Chicago Chicago Illinois USA

**Keywords:** cognitive activities, cognitive decline, cognitive resources, ethno‐racial, Latino, non‐Latino Black, non‐Latino White, older adults, race and ethnicity

## Abstract

**INTRODUCTION:**

Little is known about whether the cognitive benefits of cognitive activities and resources differ across ethno‐racial groups in late‐life.

**METHODS:**

Participants were 1702 non‐Latino White, 766 non‐Latino Black, and 324 Latino adults (≈ 76 years; *N* = 2792). Linear mixed‐effects models tested interaction by ethno‐racial group in the associations of lifespan (past and current) cognitive activity and total (past only) cognitive resources, respectively, with change in global cognition (GC) and five cognitive domains (≈ 8 ± 5 years).

**RESULTS:**

Significant interactions were observed: higher lifespan cognitive activity predicted slower decline in GC (but not in domains) among non‐Latino White participants (estimate = 0.01, standard error [SE] = 0.01) and faster decline in GC (including working memory and perceptual speed domains) among Latino participants (estimate = −0.02, SE = 0.01); no association was observed among non‐Latino Black participants. Total cognitive resources did not predict cognitive decline.

**DISCUSSION:**

Findings highlight ethno‐racial differences in the association between lifespan cognitive activity and decline in global cognition among older adults.

## Highlights

- Cognitive activity predicted slower cognitive decline in non‐Latino White adults.
- Cognitive activity predicted faster cognitive decline in Latino adults.
- Cognitive activity did not predict cognitive change in non‐Latino Black adults.
- Cognitive resources were not associated with cognitive decline in any group.

## 1 BACKGROUND

Ethno‐racial differences in the burden of Alzheimer's disease (AD) and AD‐related dementias (ADRD) persist in the United State. Compared to older non‐Latino White adults, older non‐Latino Black and Latino adults experience a higher prevalence and risk of AD/ADRD.^1^ In the absence of effective disease‐modifying treatments, it is critical not only to identify modifiable protective factors associated with slower cognitive decline but also to determine whether their effects are consistent across populations at higher risk for AD/ADRD.^1^, ^2^ Engagement in cognitively stimulating activities (e.g., reading books or visiting museums) has been proposed as a promising protective factor and contributor to cognitive reserve by helping maintain efficiency in underlying neural systems, enhancing adaptation to age‐related neuropathologic changes, or both.^3^, ^4^, ^5^ In longitudinal studies, we^6^, ^7^, ^8^, ^9^, ^10^ and others^11^, ^12^, ^13^, ^14^, ^15^, ^16^, ^17^, ^18^ have shown that more frequent participation in cognitively stimulating activities is associated with slower decline in global cognition (GC) and specific domains, such as memory and perceptual speed,^6^, ^7^, ^11^, ^12^, ^13^, ^14^, ^15^, ^16^, ^17^ as well as reduced dementia risk.^6^, ^8^, ^9^, ^10^, ^18^

Most of this evidence, however, is derived predominantly from samples of older non‐Latino White adults and studies that include more diverse populations often adjust for ethno‐racial group rather than examine potential differences across groups.^11^, ^13^, ^14^, ^17^ To our knowledge, only one longitudinal study has explicitly examined such differences. In that study, a 1‐point increase in late‐life cognitive activity was associated with a 14% slower rate of cognitive decline among older non‐Latino White adults and an 8% slower rate among non‐Latino Black adults; however, Latino participants were not included.^13^ While prior studies support the cognitive benefits of engagement in cognitive activities, particularly during late‐life,^11^, ^13^, ^14^, ^16^, ^17^ it remains unclear whether other life stages, such as childhood, young adulthood, or middle age, may represent sensitive periods for cognitive enrichment across ethno‐racially diverse populations.^19^, ^20^ Together, these gaps highlight the need for a life‐course approach to better understand how cognitively enriching experiences shape cognitive aging across diverse populations.

In addition to engagement in cognitive activities across the life course, access to cognitive resources in the home (e.g., books, newspaper subscriptions, or library cards) may provide opportunities for participation in cognitively stimulating activities. We previously found that greater availability of cognitive resources in the home during childhood and middle age is associated with higher levels of cognitive function across several ethno‐racial groups, including older non‐Latino White, non‐Latino Black, and Latino adults.^21^, ^22^ However, our prior work either focused exclusively on non‐Latino Black adults^21^ or included relatively small samples of Latino participants^22^ (*n* = 81), and both studies were cross‐sectional, precluding examination of longitudinal associations. Consequently, little is known about whether access to home‐based cognitive resources is associated with cognitive decline among ethno‐racially diverse populations of older adults.

To address these gaps, we evaluated the associations of cognitive activity and access to cognitive resources, respectively, with levels of and annual rates of change in GC and five cognitive domains (episodic memory, semantic memory, working memory, perceptual speed, and visuospatial ability) in a sample of older non‐Latino White, non‐Latino Black, and Latino adults. We hypothesized that more frequent engagement in cognitive activity and greater access to cognitive resources would each be associated with higher levels of cognition and slower rates of cognitive decline. We further hypothesized that these associations would differ across ethno‐racial groups because opportunities for engagement in cognitively stimulating activities, access to educational and cognitive resources, and exposure to broader structural and social determinants of health vary across populations and may influence how cognitive activity contributes to cognitive reserve and cognitive aging trajectories.^23^ Clarifying these relationships may inform interventions aimed at increasing engagement in cognitively stimulating activities across the life course to promote cognitive health in late‐life among diverse populations.

**RESEARCH IN CONTEXT**

1. **Systematic review**: The authors reviewed the literature to identify studies examining cognitive activities, cognitive resources, and late‐life cognitive outcomes across ethno‐racial groups. Prior work shows that greater engagement in cognitive activities over the lifespan is associated with slower cognitive decline in older non‐Hispanic White adults, but few studies have evaluated whether these associations differ across older non‐Latino White, non‐Latino Black, and Latino adults.
2. **Interpretation**: This study contributes new evidence suggesting the cognitive benefits of cognitive activities are not uniform across ethno‐racial groups. Greater engagement predicted slower decline in global cognition only among older non‐Latino White adults; this association was not observed for non‐Latino Black or Latino participants. Access to cognitive resources in the home was not associated with cognitive decline in any group.
3. **Future directions**: Future research should clarify the sociocultural, educational, and structural determinants shaping the role of lifespan cognitive activity on late‐life cognitive aging.

## 2 METHODS

### 2.1 Study populations

We included older adults (aged ≈ ≥ 65 years) enrolled in one of three ongoing epidemiological cohort studies of the Rush Alzheimer's Disease Center (RADC): the Rush Memory and Aging Project (MAP, 1997 to present),^24^ the Minority Aging Research Study (MARS, 2004 to present),^25^ and the Latino Core (LATC, 2015 to present).^26^ Briefly, participants are recruited from community centers, churches, senior housing facilities, and other community‐based settings across the Chicago metropolitan area and its surrounding suburbs. Participation in each of the cohorts requires absence of known dementia at study entry and agreement to annual clinical and cognitive evaluations conducted by examiners blinded to previously collected data. Study procedures are harmonized across the three cohorts, and all evaluations are conducted by the same research team to facilitate valid comparisons. MAP participants provide consent to brain donation at death as a condition of study enrollment, whereas brain donation is optional in MARS and the Latino Core. Non‐Latino Black participants are primarily enrolled in MARS and Latino participants are primarily enrolled in the Latino Core; additionally, we leveraged the MAP cohort, which also includes both non‐Latino Black and Latino participants. Study measures are completed in the participant's preferred language (English or Spanish). the institutional review board of Rush University Medical Center Study approved the study procedures, and all participants provided written informed consent in accordance with the Declaration of Helsinki.

### 2.2 Eligible participants for analysis

At the time of these analyses, there were 3516 older participants from MAP, MARS, and the Latino Core with a complete baseline clinical evaluation. From those, we excluded 156 participants who met the criteria for dementia at the baseline clinical examination, 18 who self‐identified as “other” ethno‐racial group, 220 with missing data on lifespan cognitive activity or total cognitive resources, and 330 without a first complete annual follow‐up visit (93 died before the first follow‐up visit; 237 were recently recruited and had not yet completed their first follow‐up visit). This resulted in a final analytical sample of 2792 participants: 1702 non‐Latino White (100% from MAP), 766 non‐Latino Black (672 [88%] MARS; 94 [12%] MAP), and 324 Latino participants (236 [73%] Latino Core; 86 [27%] MAP; 2 [< 1%] MARS).

To evaluate whether the selected participants from MAP, MARS, and the Latino Core were different from those excluded, we compared key characteristics between our analytic sample (*n* = 2792) and excluded sample (*n* = 724). We found that our analytic sample was generally similar to the excluded participants. For example, mean education was 14.7 years in the analytic sample and 14.3 years among excluded participants, 76% of the analytic sample and 72% of excluded participants were females, and mean age at study entry was 77.5 years in the analytic sample and 78.7 years among excluded participants.

### 2.3 Cognitive activity and cognitive resources

#### 2.3.1 Cognitive activity

Lifespan (past and current) cognitive activity was assessed using a previously validated 37‐item scale.^22^, ^27^ Briefly, participants reported how often they engaged in common cognitively stimulating activities across four life stages: childhood (at ages 6 and 12; 11 items), young adulthood (age 18; 10 items), middle‐age (age 40; 9 items), and late‐life (current age; 7 items). Frequency of participation in cognitive activities was rated from 1 (once a year or less) to 5 (every day or about every day). Activities varied by life stage and included visiting a library, reading a book, and attending a play (see Table S1 in supporting information for full list of items). For each life stage, the item scores were averaged to yield four separate subscale scores (each ranged from 1 to 5). A composite total score (i.e., lifespan cognitive activity) was derived by averaging the four subscale scores (range: 1–5). Higher scores reflect more frequent participation in cognitive activities.

#### 2.3.2 Cognitive resources

Past home‐based cognitive resources were assessed using a previously validated 16‐item scale measuring the presence of materials that support cognitive activities in the home.^21^, ^27^ Participants indicated (1 = yes; 0 = no) whether they had access to seven items supportive of a cognitively active lifestyle (e.g., library card and magazine subscription), and estimated the number of books in the home during childhood (age 12) and middle‐age (age 40; see Table S2 in supporting information for full list); items did not vary by life stage. Responses for questions on the number of books were rescaled to range from 0 to 1. For each life stage, the number of available items were summed to yield two subscale scores (each ranged from 0 to 8). A composite total score (i.e., total cognitive resources) was computed by adding items across both stages (range: 0–16). Higher scores indicate greater access to cognitive resources in the home.

### 2.4 Cognitive function

During annual evaluations, all participants across cohort studies completed the same cognitive protocol following identical procedures as previously detailed.^24^, ^26^, ^28^ Tests assessed the following five cognitive domains: episodic memory (word list recall and recognition, and immediate and delayed story recall tests), semantic memory (confrontational naming, verbal fluency, and word reading tests), working memory (digit span and digit ordering tests), perceptual speed (Stroop color naming and reading, symbol digit modalities, and number comparisons tests), and visuospatial ability (line orientation and progressive matrices tests). To yield a composite measure for each cognitive domain, we converted raw scores from each test into *z* scores (using the baseline mean and standard deviation from the combined parent cohorts) and averaged the *z* scores. To yield a GC score, the raw scores from the 19 tests were converted to *z* scores and averaged. Psychometric properties of these scores have been previously well established.^29^, ^30^, ^31^

### 2.5 Covariates

Covariates were assessed at the annual study evaluation concurrent with the first assessments of cognitive activity and cognitive resources (i.e., analytic baseline), including sex, age, ethno‐racial group (non‐Latino White, non‐Latino Black, or Latino), years of formal education, mode of cognitive assessment (in‐person or via telephone during the COVID‐19 pandemic), preferred language for testing (English or Spanish), and nativity (US‐born [50 United States or the District of Columbia] or non–US born). Depressive symptoms were assessed using a modified 10‐item version of the Center for Epidemiologic Studies Depression (CES‐D) scale with a higher score reflecting a greater number of symptoms reported (range: 0–10).^32^ Number of vascular disease risk factors is a continuous score based on the number of positive self‐reported factors, including hypertension, diabetes, and smoking history (range: 0–3).

### 2.6 Statistical analysis

Descriptive statistics were estimated at baseline, and differences in characteristics across ethno‐racial groups (non‐Latino White, non‐Latino Black, and Latino) were evaluated using analysis of variance and chi‐squared (*χ* ^2^) tests. Pearson correlations were computed among exposure variables and education. In a first step, we tested whether there was evidence of effect modification by ethno‐racial group in the association between lifespan cognitive activity and change in GC by including a three‐way interaction term (“exposure” × “ethno‐racial group” × “time”) in a linear mixed effects model adjusted for sex, age at baseline, years of education, language of interview (on both the intercept and slope), and mode of cognitive assessment; within‐participant correlation was captured by correlated random intercepts and slopes. Global *p* values for interaction were obtained using multivariate Wald tests. The same modeling approach was applied to test effect modification by ethno‐racial group in the association between total cognitive resources and change in GC. The Wald test for the three‐way interaction was statistically significant for both lifespan cognitive activity and total cognitive resources (Wald test *p *= 0.001 and *p *= 0.026, respectively; Figure S1 in supporting information). Given that coefficients from three‐way interaction models are not directly interpretable, we stratified all analyses by ethno‐racial group. This approach allowed us to include Latino‐specific covariates in the models, while facilitating the interpretation of the findings.

In the next step, for each ethno‐racial group, linear mixed effects models were used to evaluate the associations of lifespan cognitive activity and total cognitive resources, separately, with each cognitive outcome (GC and five cognitive domains). The base model was adjusted for age, sex, education, and language of interview (for Latino participants only). The fully adjusted model further included depressive symptoms, number of vascular disease risk factors, and nativity (for Latino participants only). Because results were similar in the base and fully adjusted models (results not shown), we present results only from the fully adjusted model. Finally, all cognitive activity subscales (childhood, young adulthood, middle‐age, and late‐life) were entered simultaneously into fully adjusted linear mixed effect models to examine which specific life stage(s) were related to cognitive outcomes regardless of all other life stages. Comparable models were fit for the cognitive resources subscales (childhood and middle‐age). In sensitivity analyses, to assess whether our findings were robust to adjustment for multiple comparisons, we repeated the regression models applying false discovery rate (FDR) correction.

Analyses were conducted in SAS/STAT software version 9.4 (SAS Institute Inc.) and R software version 4.0.3 (R Foundation for Statistical Computing) using complete case analysis. For linear mixed models, we used the hlme function of lcmm R package version 1.7.8.^33^ Statistical significance was set at *p* < 0.05, two sided. Data can be requested at the RADC Research Resource Sharing Hub at: www.radc.rush.edu.

## 3 RESULTS

### 3.1 Participant characteristics and bivariate correlations

Most participants were female (76%) across all ethno‐racial groups (Table 1). Mean age at baseline was ≈ 81 years for non‐Latino White participants, 73 years for non‐Latino Black participants, and 72 years for Latino participants. Mean years of formal education were lowest among Latino participants (11 years, standard deviation = 5) compared to non‐Latino White and non‐Latino Black participants. Most Latino participants were born outside the United States (77%) and preferred Spanish for testing (68%). Latino participants also reported the lowest levels of lifespan cognitive activity and total cognitive resources (frequencies of scale items are shown in Tables S1–S2). Non‐Latino White participants had the highest baseline global and domain‐specific cognitive scores. Correlations among cognitive activity and cognitive resources (lifespan/total and subscales) were generally consistent across ethno‐racial groups. However, among Latino participants, education showed stronger correlations (*r* ≥ 0.47) with all cognitive activity and cognitive resources variables except late‐life cognitive activity (Table S3 in supporting information).

**TABLE 1:** Baseline characteristics of participants, overall and by ethno‐racial group.

Characteristics	All	Non‐Latino White	Non‐Latino Black	Latino	*p* value^*^
	*n* = 2792	*n* = 1702	*n* = 766	*n* = 324	
Female, %	76	75	77	77	0.2
Age, years	77.5 (7.7)	80.5 (6.8)	73.1 (6.2)	71.8 (6.9)	< 0.0001
Education, years	14.7 (3.7)	15.4 (3.1)	14.8 (3.3)	10.7 (5.1)	< 0.0001
Follow‐up, years	7.8 (5.0)	7.6 (4.9)	9.1 (5.6)	6.3 (3.3)	< 0.0001
Language at the interview, %					
*English*	92	100	100	32	< 0.0001
*Spanish*	8	0	0	68	
Nativity group, %					
*Born in the US*	88	97	99	23	< 0.0001
*Not born in the US & ≥ 10 years in the US*	11	3	1	74	
*Not born in the US & < 10 years in the US*	1	0	0	3	
Depressive symptoms, median (IQR)	1 (0;2)	0 (0;1)	1 (0;2)	1 (0;3)	< 0.0001
No. of vascular disease risk factors^†^	1.2 (0.8)	1.0 (0.8)	1.5 (0.9)	1.3 (0.9)	< 0.0001
Cognitive function, *z*‐scores					
GC	0.09 (0.55)	0.20 (0.54)	−0.03 (0.51)	−0.23 (0.55)	< 0.0001
*Episodic memory*	0.10 (0.66)	0.15 (0.69)	0.08 (0.59)	−0.06 (0.61)	< 0.0001
*Semantic memory*	0.10 (0.73)	0.23 (0.68)	−0.05 (0.74)	−0.17 (0.80)	< 0.0001
*Working memory*	0.06 (0.78)	0.25 (0.71)	−0.05 (0.72)	−0.69 (0.79)	< 0.0001
*Perceptual speed*	0.08 (0.76)	0.19 (0.75)	−0.05 (0.73)	−0.15 (0.81)	< 0.0001
*Visuospatial ability*	0.06 (0.82)	0.31 (0.74)	−0.31 (0.81)	−0.31 (0.80)	< 0.0001
Cognitive activity scores (possible ranges 1–5)					
*Lifespan*	3.1 (0.6)	3.2 (0.5)	3.1 (0.6)	2.5 (0.6)	< 0.0001
*Childhood*	3.0 (0.7)	3.0 (0.7)	3.2 (0.7)	2.5 (0.8)	< 0.0001
*Young adulthood*	3.0 (0.7)	3.1 (0.7)	3.1 (0.7)	2.4 (0.8)	< 0.0001
*Middle‐age*	3.2 (0.7)	3.3 (0.6)	3.3 (0.6)	2.7 (0.8)	< 0.0001
*Late‐life*	3.1 (0.7)	3.2 (0.7)	2.9 (0.6)	2.5 (0.7)	< 0.0001
No. of cognitive resources in the home					
*Total* (possible range 0–16)	9.9 (3.3)	10.6 (2.9)	10.0 (3.1)	6.5 (3.8)	< 0.0001
*Childhood* (possible range 0–8)	4.0 (2.2)	4.5 (2.0)	3.8 (2.2)	2.0 (2.2)	< 0.0001
*Middle‐age* (possible range 0–8)	5.9 (1.7)	6.1 (1.6)	6.2 (1.6)	4.5 (2.2)	< 0.0001

*Note*: Values are expressed as mean (standard deviation), unless otherwise specified. Lifespan cognitive activity score includes childhood (ages 6 and 12; 11 items), young adulthood (18 years; 10 items), middle‐age (40 years; 9 items), and late‐life (current age; 7 items). Total cognitive resources include childhood (age 12) and middle‐age (age 40).

Abbreviations: ANOVA, analysis of variance; GC, global cognition; IQR, interquartile range; US, United States.

^*^ *p* values from ANOVA test for quantitative variables and chi‐square d(*χ* ^2^) tests for binary and categorical variables.

^†^ Vascular disease risk factors include self‐reported hypertension, diabetes, and smoking (current or former).

### 3.2 Associations of cognitive activity with cognitive outcomes by ethno‐racial group

Among non‐Latino White participants, higher lifespan cognitive activity was linked to higher levels of GC and all domains (Table 2; boldface values indicate statistical significance before correction for multiple testing). In subscale analyses, there were differential level associations of cognitive activity at each life stage with cognition. Specifically, young adulthood and late‐life activity were associated with higher GC and most domains (except episodic memory for young adulthood activity); childhood and middle‐age activity were associated with lower perceptual speed; and childhood activity was also associated with lower visuospatial ability. Longitudinally, two associations were observed: higher lifespan activity was associated with slower decline in GC and late‐life activity with slower decline in episodic memory.

**TABLE 2:** Multivariable‐adjusted mean differences in baseline level and change for GC and cognitive domains, according to frequency of cognitive activity (lifespan and at each life stage) by ethno‐racial group.

	Lifespan		Childhood (at ages 6 & 12)		Young adulthood (at age 18)		Middle‐age (at age 40)		Late‐life (at ages ≥ 65)	
Outcome	Level	Change	Level	Change	Level	Change	Level	Change	Level	Change
**Non‐Latino White (*n* = 1,702)**										
GC	**0.22 (0.02)** ^****^	**0.01 (0.01)**	−0.01 (0.02)	−0.002 (0.01)	**0.08 (0.03)** ^**^	−0.0003 (0.01)	0.02 (0.03)	0.01 (0.01)	**0.15 (0.02)** ^****^	0.004 (0.01)
Episodic memory	**0.24 (0.03)** ^****^	**0.01 (0.01)**	0.04 (0.03)	−0.01 (0.01)	0.06 (0.04)	−0.0003 (0.01)	0.05 (0.03)	0.01 (0.01)	**0.10 (0.03)** ^***^	**0.01 (0.01)**
Semantic memory	**0.27 (0.03)** ^****^	0.01 (0.01)	−0.02 (0.03)	0.01 (0.01)	**0.09 (0.03)** ^*^	−0.003 (0.01)	0.04 (0.03)	0.01 (0.01)	**0.19 (0.03)** ^****^	0.003 (0.01)
Working memory	**0.20 (0.03)** ^****^	0.004 (0.01)	−0.03 (0.03)	−0.005 (0.01)	**0.11 (0.04)** ^**^	0.001 (0.01)	0.05 (0.03)	0.004 (0.01)	**0.08 (0.03)** ^**^	0.01 (0.004)
Perceptual speed	**0.23 (0.03)** ^****^	0.01 (0.01)	**−0.09 (0.03)** ^*^	0.001 (0.01)	**0.08 (0.04)** ^*^	−0.003 (0.01)	**−0.08 (0.03)**	0.004 (0.01)	**0.40 (0.03)** ^****^	0.01 (0.01)
Visuospatial ability	**0.17 (0.03)** ^****^	0.01 (0.01)	**−0.06 (0.03)**	0.01 (0.01)	**0.13 (0.03)** ^***^	−0.004 (0.01)	0.02 (0.03)	0.004 (0.01)	**0.08 (0.03)** ^**^	0.002 (0.01)
**Non−Latino Black (*n* = 766)**										
GC	**0.11 (0.03)** ^**^	−0.01 (0.01)	−0.04 (0.03)	−0.01 (0.01)	**0.12 (0.04)** ^**^	0.001 (0.01)	0.01 (0.04)	−0.001 (0.01)	0.02 (0.03)	0.002 (0.01)
Episodic memory	**0.11 (0.04)** ^**^	−0.01 (0.01)	0.01 (0.03)	−0.01 (0.01)	0.03 (0.04)	0.001 (0.01)	0.07 (0.04)	−0.003 (0.01)	0.01 (0.04)	0.003 (0.01)
Semantic memory	**0.17 (0.05)** ^**^	−0.02 (0.01)	−0.04 (0.04)	−0.01 (0.01)	**0.19 (0.05)** ^**^	−0.01 (0.01)	−0.03 (0.05)	−0.0001 (0.01)	0.05 (0.04)	0.001 (0.01)
Working memory	0.04 (0.05)	−0.003 (0.01)	**−0.12 (0.04)** ^*^	−0.01 (0.01)	**0.22 (0.06)** ^***^	−0.01 (0.01)	−0.06 (0.05)	0.001 (0.01)	−0.01 (0.04)	0.01 (0.01)
Perceptual speed	**0.13 (0.04)** ^**^	−0.01 (0.01)	−0.08 (0.04)	**−0.01 (0.01)**	**0.17 (0.05)** ^**^	0.004 (0.01)	−0.04 (0.05)	0.0004 (0.01)	**0.09 (0.04)**	−0.0003 (0.01)
Visuospatial ability	0.03 (0.05)	0.002 (0.01)	**−0.14 (0.04)** ^*^	0.001 (0.01)	**0.16 (0.06)** ^**^	−0.0004 (0.01)	0.07 (0.06)	−0.003 (0.01)	−0.07 (0.04)	0.004 (0.01)
**Latino (*n* = 324)**										
GC	**0.17 (0.05)** ^**^	**−0.02 (0.01)**	0.03 (0.04)	−0.01 (0.01)	0.08 (0.05)	**−0.02 (0.01)**	0.002 (0.05)	0.01 (0.01)	**0.09 (0.04)**	−0.01 (0.01)
Episodic memory	0.11 (0.06)	−0.01 (0.01)	−0.01 (0.05)	−0.003 (0.01)	0.01 (0.06)	−0.02 (0.01)	−0.003 (0.05)	0.02 (0.01)	**0.15 (0.05)** ^*^	−0.01 (0.01)
Semantic memory	**0.15 (0.07)**	**−0.02 (0.01)**	0.003 (0.06)	−0.01 (0.01)	0.07 (0.07)	−0.01 (0.01)	0.02 (0.06)	0.01 (0.01)	0.08 (0.06)	−0.004 (0.01)
Working memory	**0.21 (0.07)** ^**^	**−0.02 (0.01)**	0.11 (0.06)	**−0.02 (0.01)**	**0.15 (0.07)**	−0.004 (0.01)	−0.09 (0.06)	0.01 (0.01)	0.01 (0.06)	−0.003 (0.01)
Perceptual speed	**0.25 (0.07)** ^**^	−0.02 (0.01)	−0.03 (0.06)	−0.01 (0.01)	0.12 (0.07)	−0.01 (0.01)	0.04 (0.07)	0.01 (0.01)	**0.16 (0.06)** ^*^	0.002 (0.01)
Visuospatial ability	0.10 (0.07)	−0.01 (0.01)	0.05 (0.06)	0.0001 (0.01)	−0.02 (0.07)	−0.01 (0.01)	0.05 (0.06)	−0.002 (0.01)	0.01 (0.06)	0.01 (0.01)

*Note*: Values are expressed as estimates (standard error). Boldface values indicate statistical significance before correction for multiple testing. Two sets of linear mixed effects models are shown. First, linear mixed models tested associations of lifespan cognitive activity with each cognitive outcome (GC and five cognitive domains), separately. Second, to examine the contribution of cognitive activity at each life stage, we tested associations of all cognitive activity subscales in the same model with each cognitive outcome modeled separately. Models included terms for sex, age at baseline (continuous, years), and education (continuous, years), mode of cognitive assessment, depressive symptoms at baseline (continuous), and number of vascular disease risk factors at baseline (hypertension, diabetes, smoking history). Language of interview (Spanish/English) and nativity (United States born: yes/no) were additionally considered for Latino participants only.

Abbreviations: FDR, false discovery rate; GC, global cognition.

^*^ *p* < 0.05.

^**^ *p* < 0.01.

^***^ *p* < 0.001.

^****^ *p* < 0.0001 after FDR correction.

**FIGURE 1:**
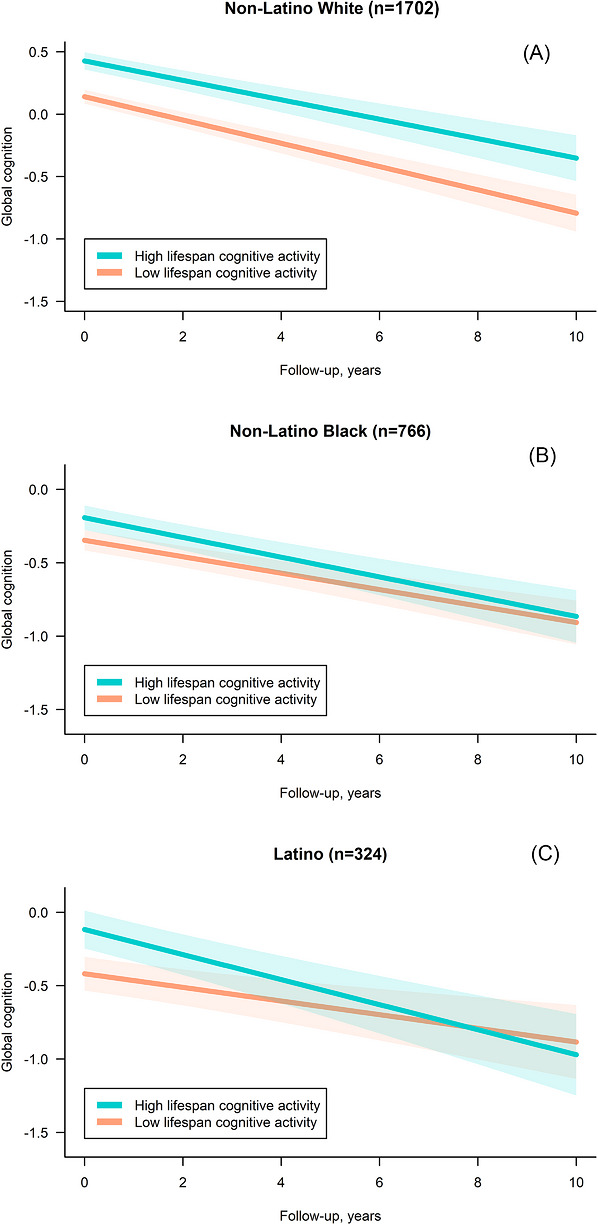
Mean estimated trajectories of global cognition by ethno‐racial group according to low and high frequency of lifespan cognitive activity: (A) non‐Latino White participants, (B) non‐Latino Black participants, and (C) Latino participants. Linear trajectories were estimated in years since baseline after controlling for the continuous score of lifespan cognitive activity, sex, age at baseline, years of education, number of depressive symptoms at baseline, number of vascular disease risk factors at baseline (on both the intercept and the slope), and mode of cognitive assessment; within‐participant correlation was captured by correlated random intercepts and slopes. Adjustment for language of interview and nativity at baseline (on both the intercept and the slope) were additionally considered for Latino participants only. Although cognitive activity was modeled as a continuous variable, we chose two levels of cognitive activity to facilitate the visualization and interpretation of our findings: 10th percentile (2.5 for non‐Latino White, 2.4 for non‐Latino Black, and 1.7 for Latino participants) versus 90th percentile (3.8 for non‐Latino White, 3.8 for non‐Latino Black, and 3.4 for Latino participants) of the cognitive activity distributions. Curves represent the marginal estimated trajectories (solid lines) with 95% confidence intervals (indicated with shading) for the most common profile of covariates for each ethno‐racial group. The choice of profile has no influence on the differences in trajectories estimated by the model.

In non‐Latino Black participants, higher lifespan cognitive activity was linked to higher levels of GC and select domains (episodic memory, semantic memory, and perceptual speed; Table 2). Similar to non‐Latino White participants, in subscale analyses, young adulthood activity was associated with higher levels of GC and most domains (except episodic memory). Additional level associations included: childhood activity with lower working memory and visuospatial ability; and late‐life activity with higher perceptual speed. Longitudinally, only childhood activity was associated with faster decline in perceptual speed. Among Latino participants, higher lifespan cognitive activity was linked to higher levels of GC and select domains (semantic memory, working memory, and perceptual speed; Table 2). In subscale analyses, late‐life activity was associated with higher levels of GC, episodic memory, and perceptual speed; and young adulthood activity with higher working memory. Longitudinally, higher lifespan activity was associated with faster decline in GC, working memory, and perceptual speed; young adulthood activity with faster decline in GC; and childhood activity with faster decline in working memory. For ease of visualization, Figure 1 displays estimated mean trajectories of GC for participants at the 10th and 90th percentiles of lifespan cognitive activity for each ethno‐racial group. Corresponding values for these percentiles were 2.5 and 3.8 among non‐Latino White participants, 2.4 and 3.8 among non‐Latino Black participants, and 1.7 and 3.4 among Latino participants.

In sensitivity analyses, after applying FDR correction, most level associations between cognitive activity and cognition remained significant (Table 2; asterisks indicate statistical significance after correction for multiple testing). There were a few exceptions. Specifically, associations between childhood activity and visuospatial ability and between middle‐age activity and perceptual speed were attenuated in non‐Latino White participants; the association between late‐life activity and perceptual speed was attenuated in non‐Latino Black participants; and associations between young adulthood and working memory and between late‐life activity and GC were attenuated in Latino participants. In contrast, all longitudinal associations between cognitive activity and cognition were no longer significant, although estimates remained in the same direction.

### 3.3 Associations of cognitive resources with cognitive outcomes by ethno‐racial group

Across all ethno‐racial groups, greater total cognitive resources were linked to higher levels of GC and all domains, except for episodic memory in non‐Latino Black and visuospatial ability in Latino participants (Table 3; boldface values indicate statistical significance before correction for multiple testing). In subscale analyses, among non‐Latino White participants, greater childhood resources were associated with higher levels of GC, working memory, and visuospatial ability, and middle‐age resources with higher GC and all domains. In non‐Latino Black participants, childhood resources were associated with higher levels of GC and all domains (except visuospatial ability), and middle‐age resources with higher visuospatial ability. In Latino participants, childhood resources were associated with higher levels of GC, semantic memory, and working memory; and middle‐age resources with GC, episodic memory, semantic memory, and perceptual speed. Longitudinally, childhood resources were associated with faster decline in perceptual speed among non‐Latino Black participants, but no other associations were observed with rates of cognitive decline.

**TABLE 3:** Multivariable‐adjusted mean differences in baseline level and change for GC and cognitive domains, according to cognitive resources present in the home (total and at each life stage) by ethno‐racial group.

	Total		Childhood (at age 12)		Middle‐age (at age 40)	
Outcome	Level	Change	Level	Change	Level	Change
**Non‐Latino White (*n* = 1702)**						
GC	**0.02 (0.004)** ^****^	0.001 (0.001)	**0.01 (0.01)**	−0.0002 (0.002)	**0.03 (0.01)** ^***^	0.003 (0.002)
Episodic memory	**0.02 (0.01)** ^**^	0.001 (0.001)	0.01 (0.01)	−0.0001 (0.002)	**0.04 (0.01)** ^**^	0.002 (0.002)
Semantic memory	**0.03 (0.01)** ^****^	0.001 (0.001)	0.01 (0.01)	−0.0005 (0.002)	**0.06 (0.01)** ^****^	0.002 (0.002)
Working memory	**0.02 (0.01)** ^****^	−0.0003 (0.001)	**0.02 (0.01)** ^*^	−0.001 (0.001)	**0.03 (0.01)** ^*^	0.0001 (0.002)
Perceptual speed	**0.02 (0.01)** ^*^	0.001 (0.001)	0.01 (0.01)	−0.001 (0.001)	**0.02 (0.01)** ^*^	0.003 (0.002)
Visuospatial ability	**0.04 (0.01)** ^****^	0.001 (0.001)	**0.04 (0.01)** ^****^	0.001 (0.001)	**0.03 (0.01)** ^**^	0.001 (0.002)
**Non‐Latino Black (*n* = 766)**						
GC	**0.02 (0.01)** ^***^	−0.001 (0.001)	**0.03 (0.01)** ^**^	−0.002 (0.002)	0.01 (0.01)	−0.001 (0.002)
Episodic memory	0.01 (0.01)	−0.0002 (0.001)	**0.02 (0.01)**	−0.001 (0.002)	−0.001 (0.01)	0.001 (0.003)
Semantic memory	**0.03 (0.01)** ^**^	−0.003 (0.001)	**0.04 (0.01)** ^**^	−0.004 (0.002)	0.01 (0.02)	−0.001 (0.003)
Working memory	**0.02 (0.01)** ^*^	−0.001 (0.001)	**0.04 (0.01)** ^**^	−0.002 (0.001)	−0.002 (0.02)	0.001 (0.002)
Perceptual speed	**0.02 (0.01)** ^**^	−0.002 (0.001)	**0.03 (0.01)** ^*^	**−0.003 (0.001)**	0.01 (0.02)	−0.0001 (0.002)
Visuospatial ability	**0.03 (0.01)** ^**^	−0.001 (0.001)	0.02 (0.01)	−0.0004 (0.001)	**0.04 (0.02)**	−0.001 (0.002)
**Latino (*n* = 324)**						
GC	**0.04 (0.01)** ^****^	−0.001 (0.002)	**0.03 (0.01)** ^*^	−0.004 (0.003)	**0.04 (0.01)** ^**^	0.002 (0.003)
Episodic memory	**0.04 (0.01)** ^***^	−0.001 (0.002)	0.02 (0.02)	−0.001 (0.003)	**0.05 (0.02)** ^**^	−0.001 (0.003)
Semantic memory	**0.05 (0.01)** ^****^	−0.004 (0.002)	**0.05 (0.02)** ^*^	−0.01 (0.004)	**0.05 (0.02)** ^**^	−0.003 (0.004)
Working memory	**0.02 (0.01)**	0.0003 (0.002)	**0.04 (0.02)**	−0.003 (0.003)	0.01 (0.02)	0.003 (0.003)
Perceptual speed	**0.04 (0.01)** ^**^	0.0001 (0.002)	0.03 (0.02)	−0.004 (0.003)	**0.05 (0.02)** ^*^	0.005 (0.003)
Visuospatial ability	0.02 (0.01)	0.0004 (0.002)	0.02 (0.02)	−0.0002 (0.003)	0.02 (0.02)	0.001 (0.002)

*Notes*: Values are expressed as estimates (standard error). Boldface values indicate statistical significance before correction for multiple testing. Two sets of linear mixed effects models are shown. First, linear mixed models tested associations of total cognitive resources with each cognitive outcome (GC and five cognitive domains), separately. Second, to examine the contribution of cognitive resources at each life stage, we tested associations of all cognitive resources subscales in the same model with each cognitive outcome modeled separately. Models included terms for sex, age at baseline (continuous, years), and education (continuous, years), mode of cognitive assessment, depressive symptoms at baseline (continuous), and number of vascular disease risk factors at baseline (hypertension, diabetes, smoking history). Language of interview (Spanish/English) and nativity (United States born: yes/no) were additionally considered for Latino participants only.

Abbreviations: FDR, false discovery rate; GC, global cognition.

^*^ *p* < 0.05.

^**^ *p* < 0.01.

^***^ *p* < 0.001.

^****^ *p* < 0.0001 after FDR correction.

In sensitivity analyses, after FDR correction, most level associations between cognitive resources and cognition remained significant (Table 3; asterisks indicate statistical significance after correction for multiple testing). There were a few exceptions. Specifically, associations between childhood resources and GC were attenuated in non‐Latino White participants; associations between childhood resources and episodic memory and between middle‐age resources and visuospatial ability were attenuated in non‐Latino Black participants; and associations between total and childhood resources with working memory were attenuated in Latino participants. Longitudinally, the association between childhood resources and perceptual speed in non‐Latino Black participants was no longer significant.

## 4 DISCUSSION

In this study of 2792 older non‐Latino White, non‐Latino Black, and Latino adults without dementia at baseline, higher lifespan cognitive activity and greater access to cognitive resources were associated with higher levels of GC and most cognitive domains across ethno‐racial groups. In longitudinal analyses, lifespan cognitive activity, but not total cognitive resources, was associated with decline in cognition. Specifically, higher lifespan activity was associated with slower decline in GC among non‐Latino White participants and faster decline in GC among Latino participants, whereas no association with cognitive change was observed among non‐Latino Black participants. Associations also varied across life stages, suggesting that the timing of cognitively stimulating activities and resources associated with late‐life cognition differed across ethno‐racial groups. In post hoc sensitivity analyses correcting for multiple testing, associations were stronger for cognitive levels than for rates of decline. Overall, these findings support a life‐course perspective in which cognitive activities and access to cognitive resources across multiple life stages appear to be associated primarily with late‐life cognitive levels rather than trajectories of cognitive decline, and that these associations vary across ethno‐racial groups.

Our study contributes to the literature on cognitive aging in several ways. First, most prior studies examining cognitive activity and cognitive decline have been conducted predominantly in samples of older non‐Latino White adults or tend to adjust for ethno‐racial group rather than explicitly evaluating potential differences across groups.^11^, ^13^, ^14^, ^17^ By directly examining effect modification by ethno‐racial group, our study responds to calls for research that evaluates whether protective factors associated with cognitive reserve and cognitive decline operate similarly across populations at higher risk for AD/ADRD. Second, we extend previous work by simultaneously examining both cognitive activity and access to cognitive resources in the home, two related but distinct dimensions of cognitive enrichment that may shape opportunities for engagement in cognitively stimulating behaviors across the life course.^22^ Third, by incorporating multiple life stages within the same analytical framework, our study extends prior research that has focused primarily on late‐life cognitive activity.^11^, ^13^, ^14^, ^16^, ^17^ Taken together, these findings advance understanding of how cognitive activity and access to cognitive resources across the life course relate to late‐life cognitive health in diverse populations.

Our finding that higher lifespan cognitive activity was associated with slower decline in GC among non‐Latino White participants is broadly consistent with prior studies linking greater engagement in cognitive or leisure activities to slower cognitive decline in predominantly non‐Latino White cohorts.^11^, ^12^, ^13^, ^14^, ^15^, ^16^, ^17^, ^18^ Unlike some previous reports,^11^, ^13^, ^16^, ^17^ however, we did not observe consistent associations between late‐life activity and decline across cognitive domains. Differences across studies could stem from variations in study populations, measures of cognitive activity, and/or model covariates, although the reasons remain unclear. We also found no association between lifespan cognitive activity and cognitive decline among non‐Latino Black participants. Although one prior study reported that greater late‐life leisure activity (e.g., watching television, listening to the radio, and reading newspapers) was associated with slower decline in this group, direct comparison is limited because the exposures differed across studies.^13^ Consequently, whether greater engagement in cognitive activities confers similar benefits for cognitive decline among older non‐Latino Black adults remains unclear.

An unexpected finding was that greater lifespan cognitive activity, driven largely by activity during young adulthood, was associated with faster decline in GC, working memory, and perceptual speed among Latino participants despite being associated with higher baseline cognitive performance. Importantly, these associations did not remain statistically significant after correction for multiple testing and therefore should be interpreted cautiously. This pattern should not be interpreted as evidence that cognitive activity increases risk of cognitive decline. Rather, it may reflect residual confounding, selective survival or attrition, measurement limitations, or contextual factors that shape how cognitive activity translates into late‐life cognitive health in this population. Latino participants reported lower levels of cognitive activity, fewer cognitive resources, and fewer years of formal education than non‐Latino White and non‐Latino Black participants, and education was more strongly correlated with cognitive activity in this group. Participation in cognitive activities and education have both been proposed as key contributors to cognitive reserve,^22^ and lower levels of lifetime cognitive engagement or educational opportunity may constrain the extent to which these experiences translate into resilience against cognitive decline.^34^ Post hoc sensitivity analyses adjusting for income and self‐reported discrimination yielded similar results, suggesting that the observed associations were not substantially explained by these indicators of socioeconomic context (Table S4 in supporting information). Nonetheless, broader structural and social determinants of health, including immigration‐related stressors, structural disadvantage, and variability in educational opportunity and quality, may shape both opportunities for cognitive engagement and trajectories of cognitive aging in older Latino adults.^34^, ^35^ Elucidating the biological and social mechanisms linking cognitive activity to cognitive decline, including pathways involving cognitive reserve, vascular health, stress‐related processes, and neuroinflammation,^36^, ^37^ remains an important direction for future research but was beyond the scope of the present study.

Across ethno‐racial groups, we also observed distinct patterns in the timing of associations among cognitive activity, cognitive resources, and cognitive performance. Cognitive activity during young adulthood was most consistently associated with higher baseline levels of GC and multiple cognitive domains among non‐Latino White and non‐Latino Black participants, suggesting that this period may represent an important but relatively understudied window in cognitive aging research. Late‐life cognitive activity was associated with higher levels of cognition among non‐Latino White and Latino participants, consistent with prior work emphasizing the potential benefits of continued cognitive engagement in older age.^11^, ^13^, ^14^, ^16^, ^17^ In contrast, childhood cognitive resources showed the most consistent associations across ethno‐racial groups, supporting the importance of early‐life opportunities for cognitive enrichment.^38^ Middle‐age cognitive resources were also associated with higher levels of cognition among non‐Latino White and Latino participants. Together, these findings reinforce the importance of a life‐course perspective on cognitive aging, suggesting that cognitively enriching environments and experiences across multiple life stages may contribute to late‐life cognitive health, although the timing of the most relevant exposures may differ across ethno‐racial groups.

This study has several limitations. First, participants were volunteers enrolled in long‐term observational studies, which may limit generalizability to the broader population of older adults. Second, cognitive activity and cognitive resources were assessed using retrospective self‐report, which may be subject to recall bias and may not fully capture the range of cognitively stimulating experiences across diverse sociocultural contexts. Third, although we adjusted for several relevant covariates and conducted sensitivity analyses adjusting for income and self‐reported discrimination, residual confounding by broader social determinants of health remains possible. Fourth, most Latino participants were born outside the United States, and our sample size did not allow us to examine heterogeneity by nativity, immigration‐related experiences, or other acculturation‐related factors. Finally, several longitudinal associations were attenuated after correction for multiple testing, underscoring the need for cautious interpretation and replication in other cohorts. Despite these limitations, our study has several important strengths. We leveraged harmonized longitudinal data from three well‐characterized cohort studies with standardized cognitive assessments, allowing us to examine both global and domain‐specific cognitive trajectories over ≈ 8 years of follow‐up. Importantly, our study includes substantial representation of older non‐Latino Black and Latino adults, populations that remain underrepresented in AD/ADRD research despite experiencing a higher burden of disease.^1^ By examining both cognitive activities and access to cognitive resources across multiple life stages, our study provides a more comprehensive life‐course perspective on cognitive enrichment than most prior work. Together, these findings highlight the importance of considering both timing and context of cognitive engagement across the life course when evaluating factors that may support cognitive health in late‐life across diverse populations.

## CONFLICT OF INTEREST STATEMENT

The authors declare no conflicts of interest. Author disclosures are available in the supporting information.

## CONSENT STATEMENT

All human subjects provided informed consent.

## Supporting information



Supporting Information: alz71487‐sup‐0001‐SuppMat.docx

Supporting Information: alz71487ȐsupȐ0002ȐDisclosuresforms.pdf
