# Supplementary material for: Associations of cognitive activity and access to resources with cognitive decline in a broad representation of older adults
Source: Alzheimers Dement. 2026 May 21;22(5):e71487. doi: 10.1002/alz.71487 (PMC13239072; doi:10.1002/alz.71487)
Supplement: Supplementary file 1 — Supporting Information: alz71487‐sup‐0001‐SuppMat.docx [file ALZ-22-e71487-s001.docx]

**SUPPLEMENTARY MATERIALS**

- **Supplementary Table 1**. Lifespan cognitive activity scale items by ethno-racial group.
- **Supplementary Table 2.** Cognitive resources scale items present in the home during childhood (at age 12) and middle-age (at age 40) by ethno-racial group.
- **Supplementary Table 3**. Bivariate correlations among education, cognitive activity (lifespan and subscales), and cognitive resources (total and subscales) by ethno-racial group.
- **Supplementary Table 4**. Sensitivity analyses additionally adjusting for income and discrimination in models examining associations of lifespan cognitive activity and total cognitive resources with global cognition by ethno-racial group.
- **Supplementary Figure 1**. Mean estimated trajectories of global cognition according to low versus high frequency of lifespan cognitive activity and total cognitive resources by ethno-racial group.

**Supplementary Table 1**. Lifespan cognitive activity scale items by ethno-racial group, mean (SD) unless specified otherwise.

| **Age** | **Scale items** | **non-Latino White**  (n=1,702) | **non-Latino Black**  (n=766) | **Latino**  (n=324) |
| --- | --- | --- | --- | --- |
| **6 y** | Play games | 3.22 (1.38) | 3.26 (1.38) | 2.79 (1.72) |
|  | Read to | 3.15 (1.55) | 3.09 (1.46) | 1.84 (1.40) |
|  | Told stories | 3.06 (1.39) | 3.19 (1.33) | 2.59 (1.55) |
| **12 y** | Time reading/day | 3.23 (1.03) | 3.41 (1.06) | 2.87 (1.25) |
|  | Visit library | 2.77 (1.06) | 2.66 (1.16) | 1.78 (1.18) |
|  | Read newspapers | 3.05 (1.63) | 3.20 (1.48) | 2.20 (1.57) |
|  | Read magazines | 2.64 (1.21) | 2.99 (1.21) | 2.48 (1.48) |
|  | Read books | 3.83 (1.25) | 3.83 (1.23) | 3.48 (1.58) |
|  | Write letters | 1.95 (0.91) | 1.95 (0.95) | 1.77 (1.10) |
|  | Play games | 3.23 (1.09) | 3.36 (1.18) | 2.54 (1.46) |
|  | Time on homework/day | 3.19 (0.97) | 3.81 (0.87) | 3.08 (1.14) |
| **Childhood subscale score** | | **3.05 (0.66)** | **3.17 (0.72)** | **2.51 (0.81)** |
| **18 y** | Visit museum | 3.26 (1.28) | 3.07 (1.34) | 2.34 (1.39) |
|  | Attend concert, play, or musicals | 3.24 (1.33) | 3.02 (1.30) | 2.36 (1.42) |
|  | Time reading/day | 3.29 (1.04) | 3.34 (1.02) | 2.46 (1.17) |
|  | Visit library | 2.93 (1.17) | 2.79 (1.25) | 1.95 (1.29) |
|  | Read newspapers | 3.58 (1.50) | 3.90 (1.24) | 2.96 (1.67) |
|  | Read magazines | 2.99 (1.19) | 3.47 (1.05) | 3.01 (1.43) |
|  | Read books | 3.68 (1.31) | 3.55 (1.30) | 2.87 (1.59) |
|  | Write letters | 2.32 (1.07) | 2.29 (1.08) | 2.04 (1.17) |
|  | Play games | 3.35 (1.12) | 2.98 (1.10) | 3.90 (1.23) |
|  | Foreign language lessons | 75% | 55% | 45% |
|  | Music lessons^*^ | 67% | 56% | 29% |
|  | Art, dance, or theater lessons^*^ | 45% | 48% | 29% |
|  | Kept a journal^*^ | 27% | 27% | 11% |
| **Young adulthood subscale score** | | **3.14 (0.65)** | **3.19 (0.70)** | **2.44 (0.82)** |
| **40 y** | Time reading/day | 3.13 (1.04) | 3.44 (1.05) | 2.56 (1.26) |
|  | Visit museum | 3.59 (1.20) | 3.35 (1.18) | 3.11 (1.36) |
|  | Attend concert, play, or musicals | 3.68 (1.25) | 3.59 (1.18) | 2.92 (1.49) |
|  | Visit library | 2.70 (1.10) | 2.39 (1.02) | 2.23 (1.20) |
|  | Read newspapers | 4.53 (0.99) | 4.56 (0.85) | 3.47 (1.60) |
|  | Read magazines | 3.58 (1.07) | 3.75 (0.98) | 3.10 (1.38) |
|  | Read books | 3.55 (1.33) | 3.43 (1.29) | 2.83 (1.46) |
|  | Write letters | 2.44 (1.09) | 2.13 (1.02) | 1.87 (1.10) |
|  | Play games | 2.75 (1.12) | 2.87 (1.06) | 2.31 (1.23) |
| **Middle-age subscale score** | | **3.36 (0.62)** | **3.33 (0.62)** | **2.73 (0.82)** |
| **≥65 y** | Time reading/day | 3.33 (1.02) | 3 (1.02) | 2.52 (1.09) |
|  | Visit library | 2.46 (1.06) | 1.97 (1.02) | 1.71 (0.95) |
|  | Read newspapers | 4.27 (1.26) | 3.99 (1.33) | 3.29 (1.57) |
|  | Read magazines | 3.47 (1.16) | 3.64 (1.15) | 3.08 (1.36) |
|  | Read books | 3.55 (1.46) | 3.11 (1.44) | 2.94 (1.52) |
|  | Write letters | 2.31 (1.11) | 1.74 (1) | 1.48 (0.83) |
|  | Play games | 3.09 (1.38) | 2.9 (1.45) | 2.53 (1.44) |
| **Lifespan score** | | **3.30 (0.66)** | **2.95 (0.65)** | **2.54 (0.70)** |

**NOTE:** Frequency of activity ranged from 1 (*least frequent*) to 5 (*most frequent*). Subscale scores reflect the mean levels of items within each life stage. Values are expressed as mean (standard deviation).

^*^ At age 18, one item (extracurricular educational experiences) was derived based on four yes or no questions asking whether participants had: 1) received instruction in a foreign language; 2) taken any music lessons; 3) taken any art, dance, or theater lessons; or 4) kept a journal. Responses were rescaled to create an extracurricular educational experiences score to range from 1 (all four questions answered as no); 2 (one question answered as yes); 3 (two questions answered as yes); 4 (three questions answered as yes); and 5 (all four questions answered as yes).

**Supplementary Table 2.** Cognitive resources scale items present in the home during childhood (at age 12) and middle-age (at age 40) by ethno-racial group.

| **Scale items** | **non-Latino White**  (n=1,702) | | **non-Latino Black**  (n=766) | | **Latino**  (n=324) | |
| --- | --- | --- | --- | --- | --- | --- |
|  | Childhood  Yes (%) | Middle-age  Yes (%) | Childhood  Yes (%) | Middle-age  Yes (%) | Childhood  Yes (%) | Middle-age  Yes (%) |
| **Subscription to daily newspaper** | 78 | 88 | 55 | 82 | 26 | 43 |
| **Subscription to magazines** | 60 | 87 | 44 | 87 | 18 | 44 |
| **Dictionary** | 90 | 99 | 84 | 99 | 52 | 90 |
| **Encyclopedia** | 57 | 77 | 53 | 89 | 30 | 69 |
| **World atlas** | 39 | 70 | 33 | 70 | 25 | 55 |
| **Globe** | 29 | 50 | 28 | 55 | 18 | 51 |
| **Library card** | 74 | 88 | 65 | 90 | 21 | 68 |
| **≥50 books** | 34 | 63 | 20 | 61 | 10 | 30 |
| **Total cognitive resources, mean (SD)** | 10.60 (2.93) | | 9.97 (3.08) | | 6.51 (3.80) | |

**NOTE:** Values represent the percentage of participants with access to each resource. Responses for the number of books in the home were recoded to a 0-1 scale before inclusion in the subscale scores. The possible total cognitive resources range is 0 to 16.

**Supplementary Table 3**. Bivariate correlations among education, cognitive activity (lifespan and subscales), and cognitive resources (total and subscales) by ethno-racial group.

|  | **1** | **2** | **3** | **4** | **5** | **6** | **7** | **8** | **9** |
| --- | --- | --- | --- | --- | --- | --- | --- | --- | --- |
| **Non-Latino White** (n=1,702) |  |  |  |  |  |  |  |  |  |
| **1.** Education | 1.00 |  |  |  |  |  |  |  |  |
| **2.** Lifespan cognitive activity | 0.40 | 1.00 |  |  |  |  |  |  |  |
| **3.** Childhood cognitive activity | 0.29 | **0.84** | 1.00 |  |  |  |  |  |  |
| **4.** Young adulthood cognitive activity | 0.39 | **0.87** | **0.67** | 1.00 |  |  |  |  |  |
| **5.** Middle-age cognitive activity | 0.36 | **0.80** | **0.49** | **0.60** | 1.00 |  |  |  |  |
| **6.** Late-life cognitive activity | 0.23 | **0.64** | 0.35 | 0.40 | **0.48** | 1.00 |  |  |  |
| **7.** Total cognitive resources | 0.34 | **0.57** | **0.51** | **0.54** | **0.45** | 0.25 | 1.00 |  |  |
| **8.** Childhood cognitive resources | 0.33 | **0.50** | **0.52** | **0.51** | 0.29 | 0.19 | **0.87** | 1.00 |  |
| **9.** Middle-age cognitive resources | 0.23 | **0.44** | 0.29 | 0.37 | **0.48** | 0.24 | **0.78** | 0.36 | 1.00 |
| **Non-Latino Black** (n=766) |  |  |  |  |  |  |  |  |  |
| **1.** Education | 1.00 |  |  |  |  |  |  |  |  |
| **2.** Lifespan cognitive activity | 0.35 | 1.00 |  |  |  |  |  |  |  |
| **3.** Childhood cognitive activity | 0.17 | **0.83** | 1.00 |  |  |  |  |  |  |
| **4.** Young adulthood cognitive activity | 0.37 | **0.89** | **0.67** | 1.00 |  |  |  |  |  |
| **5.** Middle-age cognitive activity | 0.34 | **0.80** | **0.47** | **0.66** | 1.00 |  |  |  |  |
| **6.** Late-life cognitive activity | 0.26 | **0.68** | 0.38 | **0.46** | **0.55** | 1.00 |  |  |  |
| **7.** Total cognitive resources | 0.31 | **0.57** | **0.54** | **0.55** | **0.41** | 0.25 | 1.00 |  |  |
| **8.** Childhood cognitive resources | 0.23 | **0.46** | **0.53** | **0.48** | 0.21 | 0.15 | **0.87** | 1.00 |  |
| **9.** Middle-age cognitive resources | 0.27 | **0.46** | 0.32 | **0.42** | **0.49** | 0.28 | **0.74** | 0.32 | 1.00 |
| **Latino** (n=324) |  |  |  |  |  |  |  |  |  |
| 1. Education | 1.00 |  |  |  |  |  |  |  |  |
| 2. Lifespan cognitive activity | **0.63** | 1.00 |  |  |  |  |  |  |  |
| 3. Childhood cognitive activity | **0.47** | **0.82** | 1.00 |  |  |  |  |  |  |
| 4. Young adulthood cognitive activity | **0.62** | **0.88** | **0.67** | 1.00 |  |  |  |  |  |
| 5. Middle-age cognitive activity | **0.53** | **0.84** | **0.50** | **0.68** | 1.00 |  |  |  |  |
| 6. Late-life cognitive activity | 0.35 | **0.63** | 0.33 | 0.38 | **0.54** | 1.00 |  |  |  |
| 7. Total cognitive resources | **0.58** | **0.68** | **0.57** | **0.60** | **0.61** | 0.35 | 1.00 |  |  |
| **8.** Childhood cognitive resources | **0.51** | **0.58** | **0.54** | **0.52** | **0.44** | 0.31 | **0.86** | 1.00 |  |
| **9.** Middle-age cognitive resources | **0.48** | **0.59** | **0.43** | **0.51** | **0.61** | 0.29 | **0.86** | **0.47** | 1.00 |

**NOTE:** Values represent Pearson correlation coefficients (*r*). Boldface values indicate coefficients >0.40.

**Supplementary Table 4**. Sensitivity analyses additionally adjusting for income and discrimination in models examining associations of lifespan cognitive activity and total cognitive resources with global cognition by ethno-racial group.

|  | **Model 1: Fully adjusted** | | **Model 2: Income and discrimination** | | |
| --- | --- | --- | --- | --- | --- |
|  | Level | Change | Level | Change |  |
| **Non-Latino White (Model 1: n=1,702; Model 2: n=1,549)** | | | | | |
| Lifespan cognitive activity | **0.22 (0.02)^***^** | **0.01 (0.01)**^*^ | **0.19 (0.03)^***^** | 0.007 (0.006) |  |
| Total cognitive resources | **0.02 (0.004)^***^** | 0.001 (0.001) | **0.018 (0.005)^***^** | 0.0004 (0.001) |  |
| **Non-Latino Black (Model 1: n=766; Model 2: n=749)** | | | | | |
| Lifespan cognitive activity | **0.11 (0.03)**^**^ | -0.01 (0.01) | **0.11 (0.03)^***^** | -0.008 (0.006) |  |
| Total cognitive resources | **0.02 (0.01)^***^** | -0.001 (0.001) | **0.018 (0.006)^**^** | -0.001 (0.001) |  |
| **Latino (Model 1: n=324; Model 2: n=299–301)** | | | | | |
| Lifespan cognitive activity | **0.17 (0.05)**^***^ | **-0.02 (0.01)**^*^ | **0.19 (0.05)^***^** | **-0.025 (0.009)^**^** |  |
| Total cognitive resources | **0.04 (0.01)^***^** | -0.001 (0.002) | **0.034 (0.008)^***^** | -0.001 (0.002) |  |
| **NOTE:** Values are estimates (standard error) from linear mixed-effects models. Model 1 corresponds to the fully adjusted models presented in the main analyses and includes terms for sex, age at baseline, education, depressive symptoms, number of vascular disease risk factors, and mode of cognitive assessment; language of interview and nativity were additionally included for Latino participants. Model 2 additionally adjusts for income and self-reported discrimination (where available). Sample sizes are smaller in Model 2 due to missing data on these variables. Summary measures for income and discrimination were computed among participants with available data (missing data: n = 183 [7%] for income and n = 47 [2%] for discrimination).  Indicators of socioeconomic and social context differed across ethno-racial groups in the analytic sample. The proportion reporting annual household income ≥$25,000 was 76% among non-Latino White participants, 61% among non-Latino Black participants, and 43% among Latino participants. Median perceived discrimination scores were 0 (IQR 0–1) among non-Latino White participants and 1 (IQR 0–3) among both non-Latino Black and Latino participants. | | | | |  |

**Supplementary Figure 1. Mean estimated trajectories of global cognition according to low (10^th^ percentile) versus high (90^th^ percentile) frequency of lifespan cognitive activity (upper panels; Model 1) and total cognitive resources (lower panels; Model 2) by ethno-racial group.**

Estimates are derived from two separate linear mixed models adjusted for ethno-racial group, sex, age at baseline, years of education, and language of interview (on both the intercept and the slope), and mode of cognitive assessment; within-participant correlation was captured by correlated random intercept and slope. Three-way interactions (exposure x time x ethno-racial group) were also included; the corresponding multivariate Wald-test p-values were *P*=0.001 for the model focusing on lifespan cognitive activity and *P*=0.026 for the model focusing on total cognitive resources. Curves represent the marginal estimated trajectories (solid lines) with 95% confidence intervals (indicated with shading) for the most common profile of covariates for each ethno-racial group. The choice of profile has no influence on the differences in trajectories estimated by the model.

**
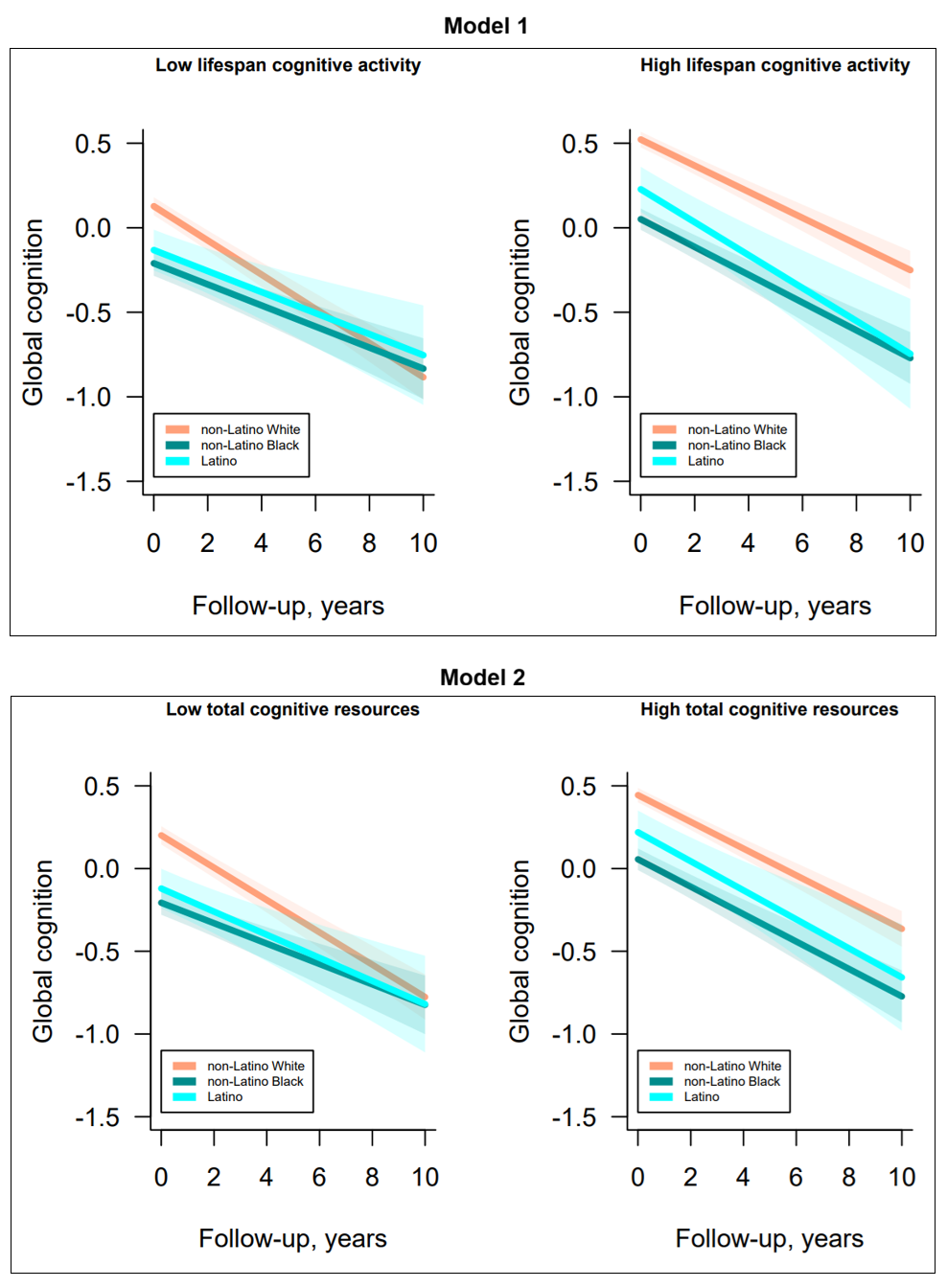
**
